# Genetic diversity of the *msp*-*1*, *msp*-*2*, and *glurp* genes of *Plasmodium falciparum* isolates in Northwest Ethiopia

**DOI:** 10.1186/s12936-018-2540-x

**Published:** 2018-10-25

**Authors:** Hussein Mohammed, Moges Kassa, Kalkidan Mekete, Ashenafi Assefa, Girum Taye, Robert J. Commons

**Affiliations:** 1 Malaria, Neglected Tropical Diseases Research Team Bacterial, Parasitic, Zoonotic Diseases Research Directorate, Ethiopian Public Health Institute, Addis Ababa, Ethiopia; 2 Menzies School of Health Research and Charles Darwin University, Darwin, Australia

**Keywords:** Genetic diversity, *Plasmodium falciparum*, Multiclonal infection, Ethiopia

## Abstract

**Background:**

Determination of the genetic diversity of malaria parasites can inform the intensity of transmission and identify potential deficiencies in malaria control programmes. This study was conducted to characterize the genetic diversity and allele frequencies of *Plasmodium falciparum* in Northwest Ethiopia along the Eritrea and Sudan border.

**Methods:**

A total of 90 isolates from patients presenting to the local health centre with uncomplicated *P. falciparum* were collected from October 2014 to January 2015. DNA was extracted and the polymorphic regions of the *msp*-*1*, *msp*-*2* and *glurp* loci were genotyped by nested polymerase chain reactions followed by gel electrophoresis for fragment analysis.

**Results:**

Allelic variation in *msp*-*1*, *msp*-*2* and *glurp* were identified in 90 blood samples. A total of 34 *msp* alleles (12 for *msp*-*1* and 22 for *msp*-*2*) were detected. For *msp*-*1* 97.8% (88/90), *msp*-*2* 82.2% (74/90) and *glurp* 46.7% (42/90) were detected. In *msp*-*1*, MAD20 was the predominant allelic family detected in 47.7% (42/88) of the isolates followed by RO33 and K1. For *msp*-*2*, the frequency of FC27 and IC/3D7 were 77% (57/74) and 76% (56/74), respectively. Nine *glurp* RII region genotypes were identified. Seventy percent of isolates had multiple genotypes and the overall mean multiplicity of infection was 2.6 (95% CI 2.25–2.97). The heterozygosity index was 0.82, 0.62 and 0.20 for *msp*-*1*, *msp*-*2* and *glurp*, respectively. There was no significant association between multiplicity of infection and age or parasite density.

**Conclusions:**

There was a high degree of genetic diversity with multiple clones in *P. falciparum* isolates from Northwest Ethiopia suggesting that there is a need for improved malaria control efforts in this region.

**Electronic supplementary material:**

The online version of this article (10.1186/s12936-018-2540-x) contains supplementary material, which is available to authorized users.

## Background

Over the last decade, the burden of malaria has declined considerably in Ethiopia, which could be the result of effective implementation of malaria control strategies at the lowest administrative levels [1]. Progress made in the reduction of this burden is attributed to implementation of effective prevention and treatment tools, such as long-lasting insecticidal nets (LLINs), indoor residual spraying (IRS) and prompt treatment of cases using artemisinin-based combination therapy (ACT) [2, 3]. However, malaria still remains one of the major health problems in the country. Approximately 60% of the population lives in malaria endemic areas [4] and malaria remains among the top ten causes of morbidity and mortality in children under 5 years [5].

The population genetic diversity of malaria varies according to the transmission intensity in malaria endemic regions and is higher in hyperendemic areas compared to areas of low endemicity [6–9]. Parasite genetic characteristics may thus reflect the dynamics of parasite transmission. Furthermore, human migration events may play a role by enhancing change in parasite populations [6]. In addition, declining malaria transmission as a result of scaling-up interventions has been shown to affect the parasite genetic diversity pattern and population structure of *Plasmodium falciparum* [7, 10], although this has not occurred in all settings. [7, 11].

Genetic diversity of *P. falciparum* is usually determined through genotyping of the polymorphic regions of the block 2 of merozoite surface protein-1 (*msp*-*1*), block 3 of merozoite surface protein-2 (*msp*-*2*) and the RII repeated region of the glutamic rich protein (*glurp*) [12]. Three major allelic families have been identified in block 2 of the *msp*-1 gene, K1, MAD20, and RO33 [13] and two allelic families in the *msp*-*2* gene, IC/3D7 and FC27 [15]. These markers are used to investigate the genetic diversity, multiplicity of infection, the level of malaria transmission, and to discriminate new from recrudescent infections in therapeutic efficacy monitoring studies [14].

To date, there has been limited assessment of genetic diversity of *P. falciparum* in Ethiopia [15, 16], despite scaling up of national malaria control interventions [17]. This study aimed to characterize the genetic diversity and allele frequencies of *msp*-*1, msp*-*2* and *glurp* genes of *P. falciparum* isolates from uncomplicated malaria patients in Humera along the Ethiopian border.

## Methods

### Study site

**Fig. 1:**
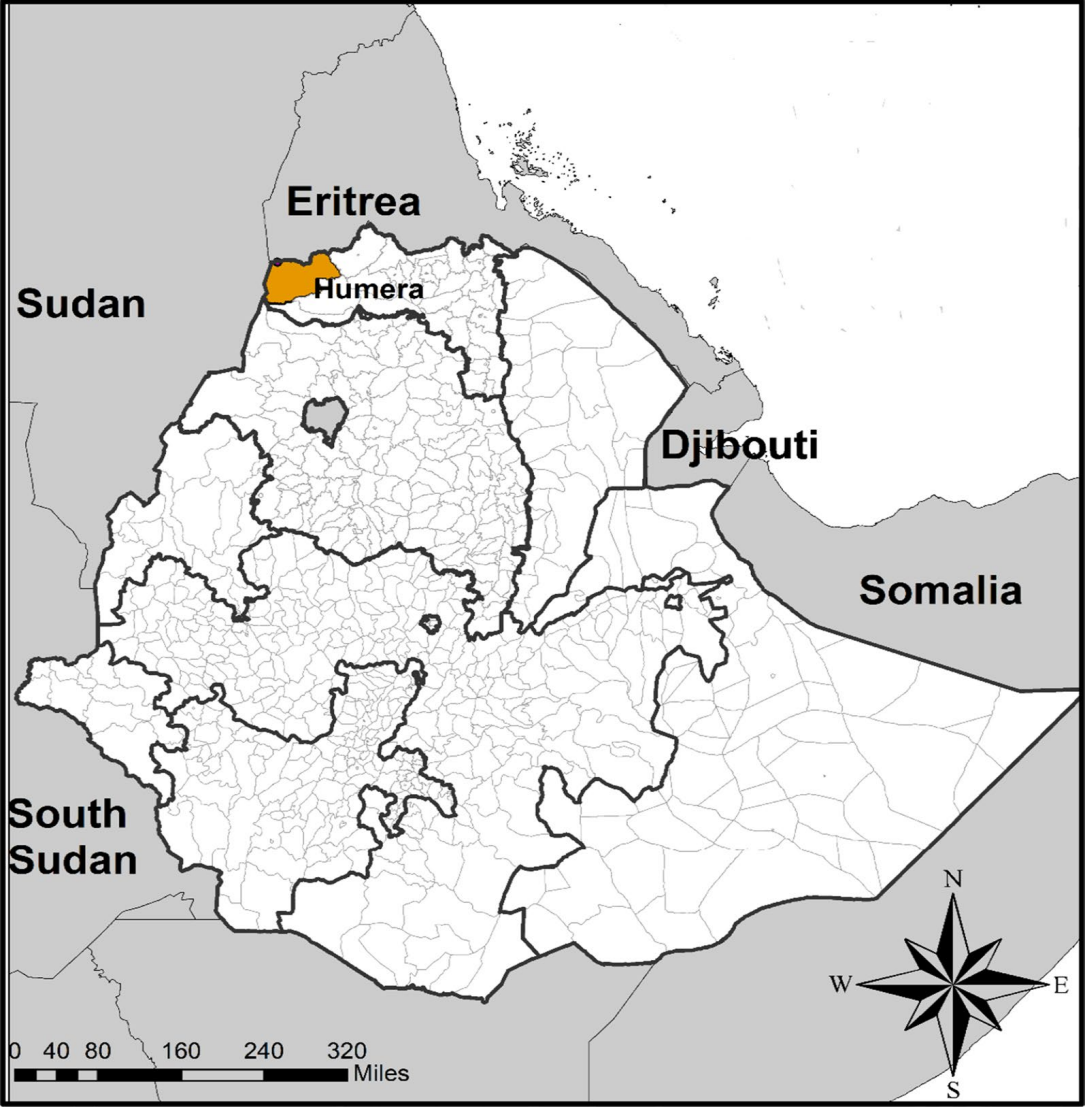
Map of the sample collection area, Humera, Northwest Ethiopia, orange colour indicated the sampling site

Samples used for this study were collected from the town of Humera, a sentinel site for monitoring of therapeutic efficacy to artemether-lumefantrine (Coartem^®^) in Northwest Ethiopia (Fig. 1). The town is situated in the Tekezze river basin and has a suitable habitat to support mosquitoes that transmit malaria. Malaria in this area is mesoendemic, with peak transmission between September and December following the rainy season [18, 19]. A migrant population flows into Humera during the harvest season (September–November), and this population bears the highest malaria burden. Sesame and barley are the main crops in the area. A detailed description of this site has been previously published [20].

### Study population and blood sample collection

A total of 90 blood spot samples were collected from patients with uncomplicated *P. falciparum* enrolled during therapeutic efficacy monitoring of artemether-lumefantrine between October 2014 and January 2015. Included patients were aged between two and 28 years, were residents within the Humera area, had presented to the local health centre with fever (axillary temperature ≥ 37.5 °C) and were found to be positive for asexual *P. falciparum*. After consent was obtained, dried blood spots were collected on day zero of enrollment through finger prick bleeding spotted onto Whatman 903^®^ filter paper (Schleicher & Schuell Bio Science, Keene, NH 03431, USA). These dried blood spots were transported and stored at − 20 °C at the Malaria Research Laboratory (Ethiopian Public Health Institute) in Addis Ababa, Ethiopia.

### Parasite DNA extraction

Parasite genomic DNA was extracted from the blood spots collected on filter papers using Chelex-100^®^ (Bio-Rad Laboratories CA) method [21], with a final volume of 200 µl for each sample and storage at − 20 °C until it was used for the amplification reaction.

### Genotyping of the *msp*-*1*, *msp*-*2* and *glurp* genes of *Plasmodium falciparum*

Nested PCR of the polymorphic regions of *msp*-*1* (block2), *msp*-*2* (block 3), and *glurp* (RII repeat region) was performed using primers and methods as previously described [22, 23]. In brief, in the initial amplification, primer pairs corresponding to conserved sequences within the polymorphic regions of each gene were included in separate reactions. The product generated in the initial amplification was used as a template in six separate nested PCR reactions. In the nested reaction, separate primer pairs targeted the respective allelic types of *msp*-*1* (K1, MAD20 and RO33), *msp*-*2* (IC3D7 and FC27), and the RII blocks of *glurp* with an amplification mixture containing 250 nM of each primer (except *glurp* nest 1 primers where 125 nM were used (Additional file 1: Table S1), 2 mM of MgCl_2_ and 125 µM of each dNTPs and 0.4 units Taq DNA polymerase. The cyclic conditions in the thermocycler (MyCycler-BioRad, Hercules, USA), for initial *msp*-*1* and *msp*-*2* PCR and initial and nested *glurp PCR* were as follows: 5 min at 95 °C, followed by 30 cycles for 1 min at 94 °C, 2 min at 58 °C and 2 min at 72 °C and final extension of 10 min at 72 °C. For *msp*-*1* and *msp*-*2* nested PCR, conditions were as follows: 5 min at 95 °C, followed by 30 cycles for 1 min at 95 °C, 2 min at 61 °C and 2 min at 72 °C and final extension of 5 min at 72 °C. The allelic specific positive control 3D7 and DNA free negative controls were included in each set of reactions [24]. Fragment analysis of *msp*-*1*, *msp*-*2* and *glurp* amplified products were then performed through electrophoresis on 2% agarose gels visualized under ultraviolet transillumination with light after staining with ethidium bromide. The size of DNA fragments were estimated by visual inspection using a 100 bp DNA ladder marker (Invitogen, Kalsruhe, Germany). The detection of a single PCR fragment for each locus was classified as an infection with one parasite genotype (monoclonal infection). Isolates with more than one genotype were considered as polyclonal infection [25]. Alleles in each family were considered the same if fragment sizes were within 20 bp intervals for *msp*-*1* and *msp*-*2* genes [26], and 50 bp intervals for *glurp* gene [12].

### Data analysis

The *msp*-*1*, *msp*-*2* and *glurp* allele frequencies were expressed as the proportion of samples containing an allelic family compared to the total number of samples that gene was detected in. Multiplicity of infection (MOI) was defined as the number of parasite genotypes per infection. Estimation of mean MOI was calculated by dividing the total number of fragments detected in *msp*-*1, msp*-*2*, or *glurp* by the number of samples in the same marker. Spearman’s rank correlation coefficient was calculated to assess associations between MOI and parasite density and age. Statistical significance was set at *P *< 0.05. The heterozygosity index (He), which represents the probability of being infected by two parasites with different alleles at a given locus, was calculated using the formula: H_e_ = [*n*/(*n* − 1)] [(1 − Σ*pi*^2^)], where *n* is the number of isolates sampled and *pi* is the allele frequency at a given locus [27]. All statistical analyses were performed using SPSS version 20.0 (SPSS Inc., Chicago, IL, USA).

### Ethical clearance

Ethical approval for the study was given by the Scientific and Ethical Review Office (SERO) of the Ethiopian Public Health Institute (EPHI). Written informed consent was obtained from parents or guardian prior to recruitment.

## Results

### Demographic and parasitological data

The characteristics of the study population are demonstrated in Table 1. The 90 patients included 56 (62.2%) males and 34 (37.8%) females, with a mean age of 15.1 ± 5.8 years. They had asexual parasitaemia ranging from 1128 parasites to 199,346 parasites/µl and a geometric mean of 14,140.9 parasites/µl (95% CI 11,047–18,078). Patients 5 to 15 years of age had the highest mean parasite density 30,429 parasites/µl (95% CI 16,104–50,159) compared to other age groups.

**Table 1:** Demographic, parasitological and clinical features of the study subjects’ profiles from Humera area, Northwest Ethiopia

Characteristic	Value
Sex ratio(M/F)	1.7 (56/34)
Age, years	
Range	2 to 28
Mean ± SD	15.1 ± 5.8
Age group	
Children (2–5 years)	3 (3.3%)
Children (5–15 years)	33 (36.7%)
Adult(≥ 15 years)	54 (60%)
Geometric mean of parasitemia (parasites/µl)	14,141 (11,046.6–18,077.9)
Parasite density range	1128–199,346

*SD* standard deviation, *µl* microliter

### Allelic diversity of *msp*-*1, msp*-*2* and *glurp* genes in *P. falciparum*

Alleles of *msp*-*1*, *msp*-*2* and *glurp* were classified according to the size of their PCR amplified fragments (Additional file 2: Figure S1; Additional file 3: Figure S2; Additional file 4: Figure S3). Successful amplification occurred in 97.8% (88/90) of samples for *msp*-*1*, 82.2% (74/90) for *msp*-*2* and 46.7% (42/90) for *glurp*.

Allele genotyping demonstrated the highly polymorphic nature of *P. falciparum* in Humera isolates with respect to *msp*-*1*and *msp*-*2*. Among *msp*-*1* isolates, three K1 (150–200 bp), five MAD20 (150–250 bp) and four RO33 (120–230 bp) allelic families were noted. The frequency of samples with only K1, MAD20, and RO33 were 1.1, 10.2 and 5.7%, respectively. The remaining 82.9% (73/88) were polyclonal infections. Among polyclonal infections carrying two allelic types, the frequency of samples with K1/MAD20, K1/RO33, and MAD20/RO33 was 21.6%, 18.2% and 25%, respectively. Infections with all three allelic types were detected in 18.2% of cases (Table 2).

**Table 2:** Genotyping of *P. falciparum msp*-*1* polymorphic region block 2 in Humera, Ethiopia

MSP-1 (n = 88)	Frequency (%)	Allele size (bp)	No of alleles	MOI
K1	1 (1.1%)	150–200	3	2.0
MAD20	9 (10.2%)	150–250	5	
RO33	5 (5.7%)	120–230	4	
K1+MAD20	19 (21.6%)			
K1+RO33	16 (18.2%)			
MAD20+RO33	22 (25%)			
K1+MAD20+RO33	16 (18.2%)			

*Bp* base pairs, *MOI* multiplicity of infection

A total of 22 different alleles were identified for *msp*-2, including ten alleles of FC27 and 12 alleles of IC/3D7. Allele sizes ranged from (250 to 700 bp) for FC27 and (280 to 780 bp) for IC/3D7 allelic families. The frequency of FC27 and IC/3D7 were 77% (57/74) and 76% (56/74), respectively. The frequency of samples with only FC27 and IC/3D7 were 24.3% and 23.0%, respectively. Thirty-nine of the isolates (52.7%) carried both *msp*-*2* allelic families (Table 3).

**Table 3:** Genotyping of *P. falciparum msp*-*2* polymorphic region block 3 in Humera, Ethiopia

*msp*-*2* (n = 74)	Frequency (%)	Allele size (bp)	No of alleles	MOI
FC27	18 (24.3%)	250–700	10	1.9
IC/3D7	17 (23.0%)	280–780	12	
FC27+IC/3D7	39 (52.7%)			

*Bp* base pairs, *MOI* multiplicity of infection

Forty-two samples were successfully genotyped for the *glurp* RII repeat region ranging from 350 to 800 bp. A total of nine different alleles were detected and coded as genotypes I-IX. Genotype III was the most common (26%), followed by genotype I (21%) and genotype IV (19%) (Table 4).

**Table 4:** Distribution of allelic variants of *glurp* RII repeat region of *P. falciparum* populations in Humera, Ethiopia

Genotypes	Allelic size variants (50 bp bin)	N (%)
I	301–350	9 (21)
II	351–400	4 (10)
III	401–450	11 (26)
IV	451–550	8 (19)
V	551–600	2 (5)
VI	601–650	1 (2)
VII	651–700	2 (5)
VIII	701–750	2 (5)
IX	751–800	3 (7)

*glurp* glutamate rich protein, *N* number of sample

**Fig. 2:**
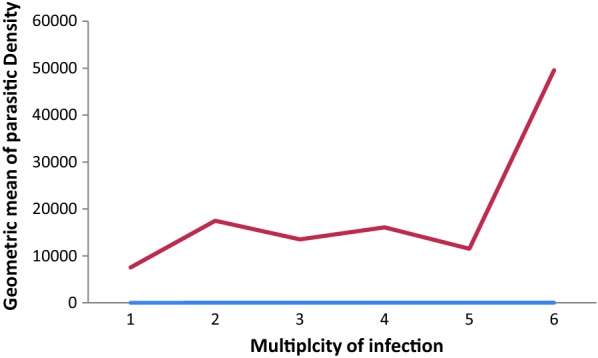
Relationship between geometric mean parasite density and multiplicity of *P. falciparum* infection

Multiple infections of *msp*-*1, msp*-*2* and *glurp* alleles were detected in 88.6% (78/88), 70.3% (52/74) and 7.1% (3/42), respectively. Sixty-three of 90 samples (70%) harboured multiple genotypes. The overall mean multiplicity of infection was 2.6 (95% CI 2.25–2.97). When considering *msp*-*1, msp*-*2* and *glurp* separately, the multiplicity of infection was 1.98 (95% CI 1.68–2.00), 1.89 (95% CI 1.68–2.28) and 1.07 (95% CI 0.99–1.15), respectively. No significant difference was observed between MOI and parasite density (Spearman rank correlation 0.198, p = 0.61; Fig. 2) or MOI and age (Spearman rank correlation 0.019, p = 0.86). The heterozygosity index was 0.82 for *msp*-*1*, 0.62 for *msp*-*2* and 0.20 for *glurp*.

## Discussion

Transmission intensity can affect the genetic diversity of the parasite population. Studies comparing areas of different transmission intensities of *P. falciparum* observe that genetic diversity is more diverse in areas of high transmission [28, 29]. Furthermore, studies in high transmission areas in Africa have shown that following enhanced malaria control interventions, genetic diversity reduces as transmission declines [10]. Therefore, understanding of the genetic structure of a parasite population can complement the implementation of malaria control interventions. No study has been conducted previously in Ethiopia using three polymorphic antigen markers, and the present study provides the most detailed assessment of genetic diversity and multiplicity of infection of *P. falciparum* isolates from Northwest Ethiopia. As Ethiopia moves towards malaria elimination, analysing the genotypes circulating along the border area is likely to be beneficial for ongoing evaluation of malaria control interventions and in understanding parasite dynamics related to imported cases.

Genotyping of *msp*-*1* and *msp*-*2* identified a higher diversity of alleles than *glurp*. This is in line with previous reports [30, 31]. Of the three allelic families of the *msp*-*1* gene, MAD20 was the predominant allelic type, similar to reports from Indonesia [32], Malaysia [33] and Sudan [34]. In contrast, in isolates from Southwest Ethiopia [15], Côte d’Ivoire and Gabon [35], K1 was the most prevalent allelic family. Variations in the prevalence of block 2 alleles between different studies likely reflect differences in geographic locations and local transmission intensity [35, 36], in a gene that is highly polymorphic and has a dynamic genetic structure that can reflect transmission pressures [37]. Allele typing of *msp*-*2* showed that the frequency of FC27 and IC/3D7 allelic families was nearly identical among the isolates; similar to findings from a study in Cambodia [24]. In agreement with previous reports [12, 38], the RII region of the *glurp* gene was also polymorphic, with nine distinct allelic fragment sizes detected.

Genetic diversity values were high according to the heterozygosity index for *msp*-*1* and moderate for *msp*-*2*. Similar genetic diversity was identified for these genes in *P. falciparum* isolates in Southwest Ethiopia from 2008 [15]. In Djibouti, a neighboring country to Ethiopia, an initially moderate level of genetic diversity declined over an 11-year period to the point that expected heterozygosity reached zero in 2009 consistent with very low diversity [39].

The present study found that nearly three-quarters of the isolates harboured multiple genotypes, with about one-quarter monoclonal. This is a similar finding to a previous study from Northwest Ethiopia that only analysed *msp*-*2* [16]. However, it is a significantly higher proportion with multiple clones than many of the locations sampled by the International Centers of Excellence for Malaria Research [9].

Population genetic studies have shown that MOI in the human population can be a proxy for transmission intensity. However, transmission intensity can also be affected by other factors such as vector biting behaviour and endemicity [9]. Inferring high transmission intensity from the presence of multiclonal infections alone has additional limitations including estimates of MOI varying by genotyping method, potential impact from sampling frequency and a non-linear relationship between MOI and transmission intensity [9]. Despite these limitations, the high levels of mixed infection in the present study, combined with evidence of moderate to high genetic diversity would be consistent with high transmission intensity in the study area. This is further supported by the presence of a high prevalence of malaria in screened patients during the study period (67%) consistent with malaria being highly endemic [20]. It is also compatible with reports from studies of African countries with intense malaria transmission in Cameroon, and Tanzania [40, 41].

High genetic diversity and MOI are evident in the present study despite the scale-up of malaria control efforts in Ethiopia. These findings are similar to reports from Sudan [11], western Kenya [42], northeastern Myanmar [43] and Nigeria [38]. Persistent genetic diversity likely reflects in part the ongoing high transmission intensity that exists in this region. While the results contrast with declining rates of genetic diversity and MOI in Djibouti and Thailand, these locations also had a reduction in malaria transmission [7, 39].

MOI has been correlated with parasite density in a number of studies, including in Congo, Brazzaville and West Uganda [26, 44]. However, similar to a study in south Benin, there was no association between MOI and parasite density in the present study [45]. This may have been due to the small number of isolates analysed. Similarly, the relationship between MOI and age remains unclear. The present study found no association between MOI and patients’ age, in agreement with studies conducted in south Benin and Senegal [45, 46], but in contrast to Central Sudan [47]. This variation may relate to differences in endemicity and the development antiparasite specific immunity. For example, there may be no association with MOI and age in regions of mesoendemicity such as the present study where immunity is similar across all ages, while there is an association in regions with intense transmission where immunity develops with age [46, 48, 49].

There is also the possibility that seasonal migration of individuals into and out of Humera contributed to the MOI and genetic diversity. Parasite gene flux mediated by human migration events is recognized to be a possible hindrance to malaria control interventions [50]. However, the current study was unable to investigate this in detail due to limits to the genotyping technique used in our study. To better understand the contribution of migration and malaria control interventions to malaria control in Ethiopia further studies on the genetic structure and diversity of *P. falciparum* in Ethiopia are needed, including across wider geographical area. In addition, use of microsatellite sequences, single nucleotide polymorphisms (SNPs) or whole-genome sequencing would provide improved insights into parasite transmission dynamics [8, 10].

Genotyping by size polymorphism is limited by processes that may lead to an inability to differentiate unique allelic fragments. Fragment size polymorphism of *msp*-*1* is under positive natural selection and alleles may converge at the population level, with fragments of the same or similar size having different sequences [13, 14]. These events may lead to an underestimation of MOI and genetic diversity, and limit the generalizability of results to other settings. However, convergence at a population level does not prevent comparison within the same population at an alternative time period, such as to assess the impact of interventions.

## Conclusions

This study reveals high genetic diversity and multiple infections in *P. falciparum* isolates from Northwest Ethiopia and is comparable to similar reports from other high transmission areas. The human population events occurring in this region may also contribute to this diversity and provide another challenge for malaria control efforts. These results suggest that additional strengthening of malaria control efforts is required. This information will serve as baseline data for future studies on dynamics of parasite transmission, and for evaluation of the effect of malaria control interventions along the Northwest border of Ethiopia.

## Additional files

**Additional file 1: Table S1.** The primers used to genotype the MSP-1, MSP2, and GLURP polymorphic regions of *P. falciparum* isolates from Humera, Northwest Ethiopia.

**Additional file 2: Figure S1.** Allele sizes and types of MSP1 allelic families.

**Additional file 3: Figure S2.** Allele sizes and types of MSP2 allelic families.

**Additional file 4. Figure S3.** Allele sizes and types of GLURP allele.

## Abbreviations

bp: basepairs
*glurp*: glutamate rich protein
MOI: multiplicity of infection
*msp*-*1*: merozoite surface protein1
*msp*-*2*: merozoite surface protein 2
PCR: polymerase chain reaction
