# Supplementary material for: Genetic diversity of the msp-1, msp-2, and glurp genes of Plasmodium falciparum isolates in Northwest Ethiopia
Source: Malar J. 2018 Oct 25;17:386. doi: 10.1186/s12936-018-2540-x (PMC6203214; doi:10.1186/s12936-018-2540-x)
Supplement: Supplementary file 1 — Additional file 1: Table S1. The primers used to genotype the MSP-1, MSP2, and GLURP polymorphic regions of P. falciparum isolates from Humera, Northwest Ethiopia. [file 12936_2018_2540_MOESM1_ESM.docx]

| **Allele** | **Primer** | **PCR round** | **Primer sequence** |
| --- | --- | --- | --- |
| Msp1 | MI- OF | Primary | 5’- CTAGAAGCTTTAGAAGATGCAGTATTG-3’ |
|  | MI- OR |  | 5’- CTTAAATAGTATTCTAATTCAAGTGGATCA-3’ |
| K1 | MI-KF | Nested | 5’- AAATGAAGAAGAAATTACTACAAAAGGTGC-3’ |
|  | MI-KR |  | 5’-GCTTGCATCAGCTGGAGGGCTTGCACCAGA-3’ |
| MAD20 | MI-MF | Nested | 5’- AAATGAAGGAACAAGTGGAACAGCTGTTAC-3’ |
|  | MI-MR |  | 5’- ATCTGAAGGATTTGTACGTCTTGAATTACC-3’ |
| RO33 | MI-RF | Nested | 5’-TAAAGGATGGAGCAAATACTCAAGTTGTTG-3’ |
|  | MI-RR |  | 5’-CATCTGAAGGATTTGCAGCACCTGGAGATC-3’ |
| Msp2 | M2-OF | Primary | 5’- ATGAAGGTAATTAAAACATTGTCTATTATA-3’ |
|  | M2-OR |  | 5’- CTTTGTTACCATCGGTACATTCTT-3’ |
| FC27 | M2-FF | Nested | 5’- AATACTAAGAGT GTAGGTGCArAT GCTCCA-3’ |
|  | M2-FR |  | 5’- TTTTATTTGGTGCATTGCCAGAACTTGAAC-3’ |
| 3D7 | M2-ICF | Nested | 5’- AGAAGTATGGCAGAAAGTAAkCCTyCTACT-3’ |
|  | M2-ICR |  | 5’- GATTGTAATTCGGGGGATTCAGTTTGTTCG-3’ |
| Glurp | G-OF | Primary | 5’- GTGGAATTGCTTTTTCTTCAACACTAA-3’ |
|  | G-OR |  | 5’- GTGGAATTGCTTTTTCTTCAACACTAA-3’ |
|  | G-OR | Nested | 5’- GTGGAATTGCTTTTTCTTCAACACTAA-3’ |
|  | G- NF |  | 5’TGTTCACACTGAACAATTAGATTTAGATCA-3’ |

Additional file 1: Table S1. The primers used to genotype the MSP-1, MSP2, and GLURP polymorphic regions of *P. falciparum* isolates from Humera, Northwest Ethiopia.
