# Supplementary figures and images for: Genetic diversity of the msp-1, msp-2, and glurp genes of Plasmodium falciparum isolates in Northwest Ethiopia
Source: Malar J. 2018 Oct 25;17:386. doi: 10.1186/s12936-018-2540-x (PMC6203214; doi:10.1186/s12936-018-2540-x)

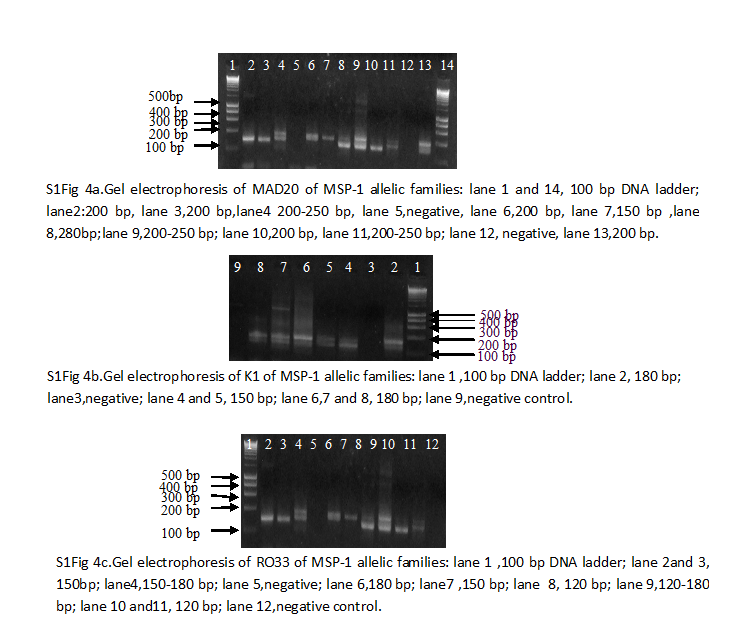

Supplement: Supplementary file 2 — Additional file 2: Figure S1. Allele sizes and types of MSP1 allelic families. [file 12936_2018_2540_MOESM2_ESM.bmp]

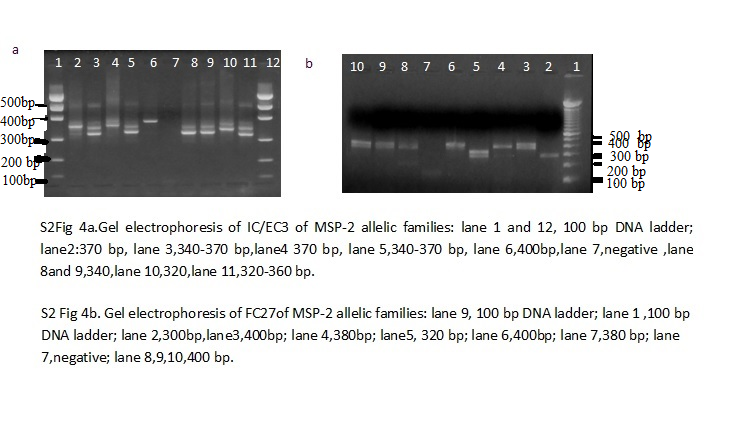

Supplement: Supplementary file 3 — Additional file 3: Figure S2. Allele sizes and types of MSP2 allelic families. [file 12936_2018_2540_MOESM3_ESM.bmp]

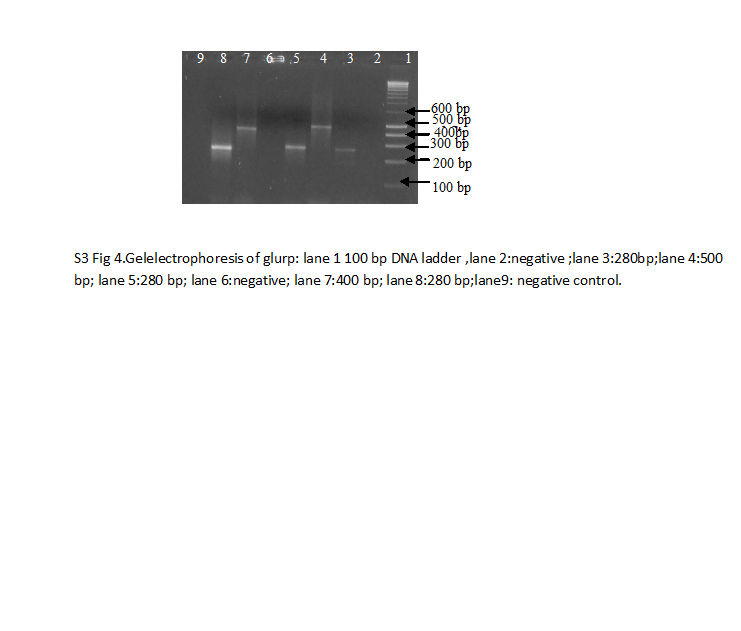

Supplement: Supplementary file 4 — Additional file 4. Figure S3. Allele sizes and types of GLURP allele. [file 12936_2018_2540_MOESM4_ESM.bmp]
